# Association of 1‐ versus 2‐week faculty rotations for resident teaching services on patient outcomes, learning environment and burnout

**DOI:** 10.1002/jhm.70389

**Published:** 2026-07-03

**Authors:** Kathryn L. Levy, Shuo Tian, Virginia Sheffield, Lauren Heidemann, Trevor Denton, Sachita Shrestha, Carla Vorndran, David Paje, Stephanie Taylor, Sarah Hartley

**Affiliations:** ^1^ Michigan Medicine, Department of Internal Medicine Ann Arbor Michigan USA; ^2^ Michigan Medicine, Department of Pediatrics Ann Arbor Michigan USA

## Abstract

**Background:**

Hospitalists increasingly staff inpatient teaching services, and many prefer shorter time‐on‐service to align with direct patient care schedules and balance competing demands. The impact of 1‐ versus 2‐week attending rotation duration on patient outcomes, faculty burnout, and learning environment is unknown.

**Objectives:**

Compare differences in patient outcomes, faculty burnout and learning environment perspectives when faculty rotate for 1‐ versus 2‐weeks on inpatient general medicine teaching services.

**Methods:**

A retrospective cohort study compared outcomes associated with 1‐ versus 2‐week attending rotations on medicine teaching services at an academic medical center. The primary outcome was hospital length of stay (LOS). Secondary outcomes included readmission within 14 days, return to the emergency department (ED) within 14 days, and death during hospitalization or within 30 days. Surveys were collected from faculty about satisfaction, burnout, and professional fulfillment, and from residents about learning environment satisfaction.

**Results:**

Of 1351 patient hospitalizations, 701 patients were cared for by faculty rounding for 1 week, and 650 by faculty rounding for 2 weeks. Median LOS was similar between the 1‐ and 2‐week groups (94 h [interquartile range [IQR], 46,166] vs. 95 h [IQR, 49,164]; *p* = .83). There were no differences in readmission, return to ED, or mortality. Faculty and resident satisfaction were similar between groups, except faculty rounding for 1 week had a mean higher satisfaction with their accessibility/availability (4.73 [SD, 0.58] vs. 4.35 [SD, 0.70]; *p* = .03). There was no difference in professional fulfillment or burnout.

**Conclusions:**

Hospitalist rotation duration of 1 versus 2 weeks on teaching services was not associated with differences in patient outcomes, overall satisfaction, or faculty professional fulfillment or burnout. This suggests that scheduling flexibility can be considered for teaching services.

## INTRODUCTION

Delivering high‐quality and efficient care to patients while creating an effective learning environment and avoiding burnout is often challenging in academic practice.^1^, ^2^ This is especially true for busy inpatient medicine teaching services, where faculty are responsible for managing acute and often complex patient care issues while attending to the needs of multiple learners at various stages of training.^3^, ^4^, ^5^ Consequently, faculty time on service has become a focus of process improvement, yet the optimal duration remains unknown.^1^, ^4^, ^5^, ^6^, ^7^, ^8^, ^9^, ^10^

In 2009, Lucas et al. compared faculty rotating for 2 weeks versus 4 weeks and found no difference in patients' unplanned revisits to the emergency department or urgent ambulatory clinics, or readmission.^4^ In this study, faculty on 2‐week rotations had lower burnout scores, but residents and medical students rated faculty lower on their ability to evaluate trainees. Anecdotally, many institutions have further shortened time‐on‐service blocks with learners to as little as 1 week. In the intensive care unit (ICU) setting, 1‐ versus 2‐week faculty rotations were not associated with differences in mortality, ICU length of stay (LOS), or duration of mechanical ventilation.^11^ To our knowledge, outcomes associated with 1‐ versus 2‐week faculty rotations for the inpatient general medicine service setting have not been compared.

The growth of hospital medicine as a field has led to more hospitalists staffing inpatient medicine teaching teams, benefitting both trainees and patient care.^3^, ^12^, ^13^ However, the traditional 2–4‐week teaching block schedule may not align with the complex demands of hospitalist schedules, which involve varying block lengths, evening and night shifts, and many faculty with non‐clinical responsibilities.^9^, ^10^, ^14^, ^15^, ^16^ No gold standard exists for an optimal scheduling model for academic hospitalists. Additionally, the inflexible structure of traditional teaching blocks may have unintended consequences for hospitalist faculty, as demanding schedules and limited autonomy over one's schedule are known drivers of burnout.^2^, ^17^ Given the growing reliance on hospitalists to staff these services, it is important to understand how faculty scheduling affects patient outcomes, faculty well‐being, and both faculty and resident satisfaction with the learning environment.

Therefore, we compared patient outcomes, faculty professional fulfillment and burnout scores, and faculty and resident satisfaction with 1‐ versus 2‐week faculty rotations on inpatient medicine teaching services. We hypothesized that there would be no difference in patient outcomes or in faculty and resident measures between 1‐week and 2‐week teaching service blocks.

## METHODS

### Setting and intervention

The study was performed at a Midwest tertiary care academic hospital between July 27, 2024, and March 25, 2025, comprising eight 4‐week resident rotations termed “blocks” at our institution. Services included four general medicine inpatient teams, each made up of one faculty, one senior resident (postgraduate year [PGY]2‐4), two interns (PGY1), and one to three medical students at any time.

Faculty rotated for either 1‐ or 2‐week blocks with transitions on Saturdays. Faculty schedules were determined through the scheduling application Qgenda (QGenda LLC) for each day of the study period from July 27, 2024, to March 7, 2025. Faculty were generally assigned to clinical service in 1‐ or 2‐week blocks based on their expressed preferences. Six faculty expressed preference for 2‐week blocks, 18 faculty expressed preference for 1‐week blocks, 21 faculty had no preference on service length, and four were hired after the preference request went out. Only two of the faculty who expressed preference were scheduled for a block length that did not match their preference, which accounted for a total of seven of the service weeks analyzed. Senior residents generally rotated in 4‐week blocks with transitions on Saturdays; but two senior residents were only scheduled for a 2‐week block. Interns generally rotated in 4‐week blocks with transitions on Mondays; but four interns were only scheduled for a 2‐week block. Resident assignment to teams followed the standard process used in the residency program based on resident requests and clinic days. Residents have a half‐day clinic approximately twice over a 4‐week period. Residents have on average 1 day off per week, which is scheduled by the senior resident.

Patient demographic and clinical data were pulled from the Epic (Verona, WI) electronic health record system. All patients admitted to the services during the study period were eligible for inclusion. Patients were categorized based on the supervising faculty's service block: those who received care from faculty rounding 1‐week blocks were assigned to the “1‐week group,” and those receiving care from faculty rounding 2‐week blocks to the “2‐week group.” Patients who received care that overlapped both 1‐week and 2‐week faculty schedules were excluded from analysis.

### Patient outcomes

The primary patient outcome was LOS. Secondary outcomes included readmission or return to the emergency department within 14 days of discharge, and death during hospitalization or within 30 days of discharge.

### Faculty and resident surveys

Non‐incentivized surveys were developed by four authors (K.L., S.H., L.H., and V.S.) who round on general medicine team and have medical education leadership roles, to capture perceptions of faculty and residents about their time on service, burnout, and professional fulfillment in faculty, and preferences of rotation duration by faculty and residents (File S2).

Along with basic demographic information and preferences on service length, the surveys included select 5‐point Likert scale questions from the VA Learner Perception Survey that were felt to be most relevant to the outcomes of interest.^18^ This included questions about clinical skills, interest in teaching, morale, degree of autonomy and supervision provided to the resident team, and time for learning and patient safety. The VA Learner Perception study was chosen because it is standardized, previously validated questions for evaluating educational interventions. In addition, the faculty survey included the Professional Fulfillment Index (PFI), a 16‐point instrument to assess physicians’ professional fulfillment and burnout.^19^ The PFI tool was chosen because it is a brief, physician‐relevant instrument which provides a balanced assessment of well‐being by measuring both positive and negative dimensions. It has demonstrated strong reliability and validity. The surveys were pilot tested to establish content validity with faculty, chief residents, and senior residents who work on medicine wards but were not scheduled during the study period. No changes to the survey questions were needed based on feedback from pilot testing.

Learners and faculty were also asked whether they preferred faculty rounding blocks of 1‐week or 2‐week duration or had no preference. They were also asked to explain their preference using free‐text responses. We analyzed the brief survey comments using inductive thematic analysis. One member of the study team (L.H.) independently reviewed all comments, conducted initial coding, and grouped into themes. One additional author (K.L.) reviewed coding structure and themes, providing feedback in discussions. Themes were refined until consensus was reached.

Surveys were distributed electronically with responses collected via Qualtrics (Provo, UT) every 2 weeks from August 14, 2024, to March 25, 2025, for faculty and every 4 weeks for residents, with reminder emails sent mid‐cycle. Near the end of the study, an additional final reminder was sent to those who had been on service in the prior 16 weeks. To account for schedule disruptions, faculty were only invited to participate if they worked for at least 5 consecutive days on service. All residents scheduled to work on these services over the study period were invited to participate. Participants were invited to complete a survey after each service rotation, allowing for multiple responses per person. Surveys less than 50% complete were excluded from analysis.

Faculty responses were grouped by whether they rounded for 1 or 2 consecutive weeks. Resident responses were grouped by the duration of their supervising faculty's rotation: 1‐week group, 2‐week group, or mixed‐week group. Mixed‐week blocks included those in which residents were supervised by both 1‐week and 2‐week faculty or experienced gaps in coverage with multiple covering faculty. These groups—1‐week, 2‐week, mixed‐week—were the groups used for analysis of the survey results to compare groups.

This study was deemed exempt from review by our Institutional Review Board (linked applications HUM00258515 for survey arm and HUM00269653 for patient outcomes arm).

### Analysis

Descriptive statistics were used to summarize patient and faculty survey characteristics by groups. Categorical variables were compared using *χ* ^2^ or Fisher's exact tests as appropriate and presented as *N* (%). Continuous variables were compared using Wilcoxon's rank‐sum test and presented as median (IQR) for patient data and mean (SD) for faculty survey data. Logistic regression analysis was performed to investigate the association of patient outcomes with different groups (2 weeks vs. 1 week) adjusted by age, sex, and CCI. All analyses were performed using RStudio software version 2024.09.0.

The PFI was scored per the developers’ recommendations by averaging the items scored in each section and using dichotomous cut‐points previously defined in the literature.^19^, ^20^ The burnout items were averaged using a cut‐point of above 1.33, meeting the threshold for burnout. The professional fulfillment items were averaged using a cut‐point of above 3.0, meeting the threshold for professional fulfillment.

## RESULTS

### Patient characteristics

A total of 1488 patient hospitalizations were identified, 1351 met the inclusion criteria, including 701 (52.9%) patients cared for by faculty rotating for 1 week and 650 (48.1%) patients cared for by faculty rotating for 2 weeks. Baseline characteristics of patients are shown in Table 1. The median age of patients was 64 years (IQR, 49–79 years), 51% were male, 77% were White, and the median Charlson Comorbidity Index was 6 (IQR, 3–9).

**Table 1:** Characteristics of patients assigned to 1‐ versus 2‐week group based on faculty time on service.

Characteristic	Overall, *N* = 1351	One‐week group, *N* = 701	Two‐week group, *N* = 650
Age, median (IQR)	64 (49, 75)	64 (47, 75)	65 (50, 75)
Sex, *N* (%)			
Female	657 (48.6)	332 (47.4)	325 (50.0)
Male	694 (51.4)	369 (52.6)	325 (50.0)
Race, *N* (%)			
White	1045 (77.4)	539 (76.9)	506 (77.8)
Black	201 (14.9)	109 (15.5)	92 (14.2)
Other/unknown	105 (7.8)	53 (7.6)	52 (8.0)
Ethnicity, *N* (%)			
Hispanic	44 (3.3)	22 (3.1)	22 (3.4)
Non‐Hispanic	1,287 (95.3)	665 (94.9)	622 (95.7)
Unknown	20 (1.5)	14 (2.0)	6 (0.9)
Ever transfer from ICU to floor, *N* (%)	42 (3.1)	27 (3.9)	15 (2.3)
Transfer from floor to ICU, *N* (%)	35 (2.6)	18 (2.6)	17 (2.6)
Charlson comorbidity index, median (IQR)	6 (3, 9)	6 (2, 9)	6 (3, 9)
Number of attendings, median (IQR)	1 (1, 2)	1 (1, 2)	1 (1, 1)

Abbreviations: ICU, intensive care unit; IQR, interquartile range.

### Patient outcomes

Patient outcomes are shown in Table 2. No differences were observed in the primary outcome, LOS, (median, 94 h [IQR, 46–166 h] for the 1‐week group versus 95 h [IQR, 49–164 h] for the 2‐week group; *p* = .83) or any of the secondary outcomes including readmission within 14 days, return to the emergency department within 14 days after discharge, or death during hospitalization or within 30 days.

**Table 2:** Outcomes of patients in the 1‐ versus 2‐week faculty rotation.

Outcome	Overall, *N* = 1351	One‐week group, *N* = 701	Two‐week group, *N* = 650	Unadjusted difference (median or rates)	95% CI	*p* value	Adjusted OR/IRR^a^	95% CI	*p* value
Hospital LOS (day), median (IQR)	4 (2, 7)	4 (2, 7)	4 (2, 7)	0	(0.0, 0.0)	.85	IRR: 0.97	(0.89, 1.06)	.51
Hospital LOS (hour), median (IQR)	94 (49, 166)	94 (49, 167)	95 (49, 164)	−1	(−8, 6)	.83	IRR: 0.97	(0.89, 1.06)	.48
Readmission in 14 days, *N* (%)	216 (16.0%)	109 (15.5%)	107 (16.5%)	−0.9%	(−5.0%, 3.2%)	.65	OR: 1.10	(0.82, 1.48)	.51
ED return in 14 days, *N* (%)	233 (17.2%)	121 (17.3%)	112 (17.2%)	0.03%	(−4.0%, 4.1%)	.99	OR: 1.03	(0.78, 1.38)	.81
Death in hospital, *N* (%)	17 (1.3%)	7 (1.0%)	10 (1.5%)	−0.5%	(−1.9%, 0.8%)	.37	OR: 1.56	(0.59, 4.33)	.37
Death in 30 days after hospital admission, *N* (%)	47 (3.5%)	30 (4.3%)	17 (2.6%)	1.7%	(0.42%, 3.7%)	.10	OR: 0.60	(0.32, 1.09)	.10

Abbreviations: CI, confidence interval; IRR, incidence rate ratio; IQR, interquartile range; LOS, length of stay; OR, odds ratio.

^a^ Calculated by logistic regression analysis adjusted by patient age, sex, and CCI.

### Faculty and resident characteristics and survey details

The faculty survey was distributed after 89 rotations, representing 49 unique faculty. Twelve responses were excluded for being less than 50% complete, and one was excluded for reporting working on a service not included in the study. Faculty response rate was 53% (47/89) with 30 in the 1‐week group and 17 in the 2‐week group. The resident survey was distributed after 99 rotations, representing 95 unique residents. Four responses were excluded for being less than 50% complete. Resident response rate was 44% (44/99) with 15 in the 1‐week group, 20 in the 2‐week group, and 9 for the mixed‐week group. Demographics of faculty and resident survey participants are available in File S2.

### Faculty and resident learning environment satisfaction

Higher satisfaction with accessibility/availability was reported on the 5‐point Likert scale by faculty on 1‐week versus 2‐week rotations (4.73 [SD, 0.58] vs. 4.35 [SD, 0.70]; *p* = .03); no other learning‐environment differences were significant (Table 3).

**Table 3:** Faculty and resident learning environment satisfaction by duration of faculty rotation.

Faculty's **survey questi**on	Overall, *N* = 47	One‐week group, *N* = 30	Two‐week group, *N* = 17	Unadjusted mean difference	95% CI	*p* value
Your clinical skills, mean (SD)	4.53 (0.58)	4.53 (0.57)	4.53 (0.62)	0.00	(−0.37, 0.38)	.96
Your interest in teaching, mean (SD)	4.57 (0.62)	4.57 (0.57)	4.59 (0.71)	−0.02	(−0.43, 0.39)	.66
Your accessibility/availability, mean (SD)	4.60 (0.65)	4.73 (0.58)	4.35 (0.70)	0.38	(0.03, 0.79)	.03
Your morale, mean (SD)	4.45 (0.85)	4.57 (0.82)	4.24 (0.90)	0.33	(−0.21, 0.87)	.11
Degree of autonomy provided to resident team, mean (SD)	4.49 (0.66)	4.50 (0.68)	4.47 (0.62)	0.03	(−0.37, 0.43)	.78
Degree of supervision provided to resident team, mean (SD)	4.45 (0.65)	4.50 (0.68)	4.35 (0.61)	0.15	(−0.24, 0.54)	.32
Patient safety, mean (SD)	4.62 (0.61)	4.67 (0.61)	4.53 (0.62)	0.14	(−0.24, 0.52)	.36

Resident's survey question	Overall, *N* = 44	One‐week group, *N* = 15	Two‐week group, *N* = 20	Mixed‐week group, *N* = 9	*p* value
Faculty's clinical skills, mean (SD)	4.75 (0.69)	4.60 (0.83)	4.80 (0.70)	4.89 (0.33)	.39
Faculty's interest in teaching, mean (SD)	4.73 (0.62)	4.60 (0.83)	4.85 (0.49)	4.67 (0.50)	.32
Faculty's accessibility/availability, mean (SD)	4.82 (0.58)	4.73 (0.80)	4.95 (0.22)	4.67 (0.71)	.38
Faculty's morale, mean (SD)	4.82 (0.54)	4.60 (0.83)	4.95 (0.22)	4.89 (0.33)	.17
Overall satisfaction with the faculty, mean (SD)	4.75 (0.69)	4.60 (0.83)	4.80 (0.70)	4.89 (0.33)	.39
Degree of autonomy, mean (SD)	4.80 (0.55)	4.67 (0.49)	4.80 (0.70)	5.00 (0.00)	.08
Degree of supervision, mean (SD)	4.75 (0.65)	4.53 (0.74)	4.80 (0.70)	5.00 (0.00)	.07
Patient safety, mean (SD)	4.82 (0.50)	4.73 (0.59)	4.85 (0.49)	4.89 (0.33)	.68
Time for learning, mean (SD)	4.30 (1.05)	3.87 (1.25)	4.45 (1.00)	4.67 (0.50)	.09
Overall satisfaction with the learning environment, mean (SD)	4.68 (0.71)	4.60 (0.83)	4.70 (0.73)	4.78 (0.44)	.89

Abbreviations: CI, confidence interval; SD, standard deviations.

### Faculty professional fulfillment

There was no difference in professional fulfillment or burnout between the two faculty groups. Faculty scored an average of 3.04 on the professional fulfillment section of the PFI with 48.9% (23/47) meeting the cut‐point (3.0) for professional fulfillment and no difference in the proportion meeting that cut‐point between faculty groups (53.3% for 1 week vs. 41.2% for 2 weeks; *p* = .55). Faculty scored an average of 0.81 on the burnout items with 21.3% (10/47) meeting the cut‐point (1.33) for burnout with no difference in proportion meeting the cut‐point between faculty groups (20.0% for 1 week vs. 23.5% for 2 weeks; *p* > .99). Further details are available in the File S2.

### Faculty and resident preferences for faculty service length

Faculty and residents report varied preferences on the duration of faculty time on service, which is shown in Figure 1. For faculty, 60.0% (18/30) of those in the 1‐week group preferred 1 week, and 82.3% (14/17) of those in the 2‐week group preferred 2 weeks. There was no obvious difference for faculty overall preference between 1‐week and 2 weeks. Among residents, 65.9% (29/44) preferred that faculty rotate for a duration of 2 weeks while 11.4% (5/44) preferred 1 week, and 22.7% (10/44) had no preference.

**Figure 1:**
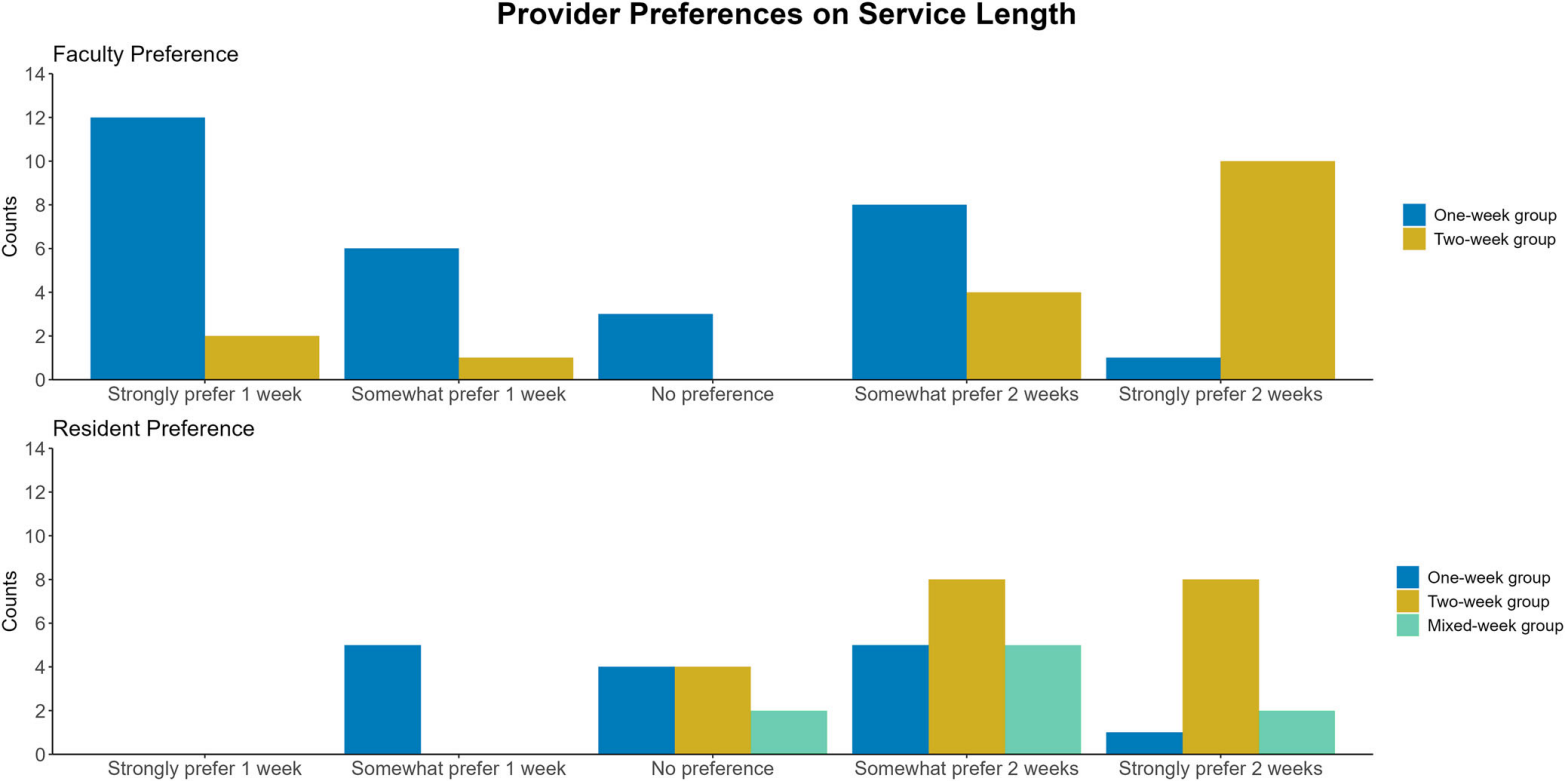
Faculty (top panel) and resident (bottom panel) preferences regarding attending physician service duration, stratified by assignment to the 1‐week, 2‐week, or mixed‐week groups.

Analysis of free‐text comments reveals several themes. Residents noted that 1‐week blocks allowed for exposure to greater variation in attending styles, which supports learning. Additionally, shorter blocks reduced the impact of mismatches in working style between learners and supervisors. Residents reported that 2‐week blocks allowed for enhanced continuity of supervision, which supported efficiency, reduced onboarding, and improved patient continuity. Residents also commented that longitudinal relationships helped build rapport and enabled tailored teaching and development.

Faculty who preferred 1‐week rotations cited that shorter rotations allowed for increased focus and energy as well as decreased burnout and fatigue. They also noted increased exposure to different learners. Lastly, many commented on the ease of balancing personal life and administrative responsibilities, as well as ease of scheduling. Faculty who preferred 2‐week blocks cited enhanced patient continuity, and building relationships, and knowing the learner. Ease of scheduling was also cited as a reason for 2‐week block preference.

## DISCUSSION

To our knowledge, this is the first study to examine differences associated with faculty rotating for 1 versus 2 weeks on inpatient teaching services. We found no observable differences in patient outcomes, faculty professional fulfillment or burnout, and only a minimal difference in perceptions of the learning environment, with faculty scheduled for 1‐week reporting greater satisfaction with their accessibility/availability. Preferences for rotation length varied among faculty and residents.

This study adds to the growing body of literature about faculty time on service and its association with patient outcomes. Prior studies on hospitalist service duration have shown mixed results regarding patient outcomes around LOS, mortality, and readmission rates.^9^, ^10^, ^16^, ^21^, ^22^, ^23^ Some studies show increased continuity with treating hospitalist is associated with decreased LOS but the data is not universal and often teaching services are not included in these studies.^10^, ^16^, ^21^, ^22^ We add to the literature by examining teaching services and had findings consistent with prior work showing no observed differences in patient outcomes.^4^ In our setting, residents typically rotate for 4 weeks, which may provide continuity that benefits patient outcomes. Alternatively, rotating supervising faculty could bring a different perspective to patient care that could lead to unmeasured benefits. Further research may assess whether resident rotation length would alter these findings.

Faculty rotating for 1 week at a time reported higher satisfaction with their accessibility/availability. While this may reflect an ability to better protect schedules from competing demands during shorter service blocks, some faculty also feel higher energy levels and the ability to focus on shorter blocks, which may tie into their sense of accessibility/availability. In contrast, those on 2‐week rotations may have roles (such as leadership or research)that are difficult to defer for the full duration of their time on service, leading to faculty trying to balance multiple competing demands and a sense of being less accessible/available to the team. Notably, quantitative data show residents did not perceive differences in faculty availability between models, highlighting a discordance between faculty and resident perceptions. This suggests that faculty experiences of accessibility/availability may not directly translate to resident perceptions of the learning environment. It is important to note that there were no direct questions asked of faculty and residents about satisfaction of continuity with team members, which was a major theme in the qualitative comments from faculty and residents who preferred 2‐week rotations. More research is needed to further understand how the perceived importance of team continuity impacts the learning environment.

It is important to ensure medical education research not only includes educational outcomes but also ties in clinical outcomes.^24^ Our study does this by pairing evaluation of patient outcomes with measures of the learning environment and faculty well‐being, including burnout and professional fulfillment. We were particularly interested in the effect on the learning environment, given concerns that reduced continuity with faculty might negatively affect learners’ perceptions and the stated expectations from the Accreditation Council for Graduate Medical Education (ACGME) that the learning environment must balance workload, provide adequate resources, and prioritize resident well‐being and patient safety.^25^ The absence of significant differences in learning environment preferences may suggest that both scheduling models are compatible with maintaining time and space for learning while providing appropriate oversight of patient care in both models.

Finally, it is reassuring that there was no difference in faculty burnout between different service lengths, and a majority of the faculty did not meet the established threshold for burnout. This could be partly driven by self‐selection of rotation duration included in our division's scheduling process. One of the biggest dissatisfiers and contributing factors to burnout when physicians are surveyed is lack of control over one's schedule.^17^ Allowing faculty agency in their work schedule contributes to one's sense of well‐being and can be used as a tool to mitigate burnout. Overall, our findings support allowing faculty autonomy in selecting rotation length, with no evidence of adverse associations with patient outcomes or learning environment.

### Limitations

This study was performed at a single institution; thus, the results may not be readily generalizable to other settings. The use of self‐reported measures and the voluntary nature of the survey could introduce information bias. Given the modest survey response and that respondents could answer more than once, both selection bias and clustering are a limitation of this study. Regarding differences between faculty rotating for 1‐week versus 2‐week blocks, there are several potential confounders, including faculty experience, gender distribution, and non‐clinical time, that we were unable to account for given the small sample size and extremely skewed outcome distribution. Additionally, select items from the VA Learner Perception Survey that were most relevant to evaluating faculty service length on an inpatient internal medicine service were used in our study, which may limit the study's overall validity due to partial representation of the full survey constructs. Self‐selection of length of time on service could also be a source of bias in survey responses, as faculty may hope to sustain the option for their preferred 1‐ or 2‐week rotation duration. Furthermore, given the anonymous nature of the survey, we could not evaluate correlations between faculty preference for scheduling and patient outcomes. Finally, granular details on resident scheduling were not evaluated, thus that impact is unknown.

## CONCLUSION

In this single institution retrospective cohort study comparing 1‐ versus 2‐week faculty rotations, we found no difference in association with patient outcomes, faculty burnout or professional fulfillment, or satisfaction with learning environment apart from increased faculty satisfaction with accessibility/availability when on 1‐week rotations. These findings suggest that flexible faculty scheduling for general medicine teaching services is a viable option without measurable differences in patient or educational outcomes.

## CONFLICT OF INTEREST STATEMENT

The authors declare no conflicts of interest.

## Supporting information

Supplement 1 ‐ Survey Questions.

Supplement 2 ‐ Faculty and Resident Demographics.

Supplement 3 ‐ PFI Results.

## Data Availability

The data that support the findings of this study are available from the corresponding author upon reasonable request.
