# Supplementary material for: Association of 1‐ versus 2‐week faculty rotations for resident teaching services on patient outcomes, learning environment and burnout
Source: J Hosp Med. 2026 Jul 3;21(11):1263–71. doi: 10.1002/jhm.70389 (PMC13728539; doi:10.1002/jhm.70389)
Supplement: Supplementary file 1 [file JHM-21-1263-s001.docx]

**Survey Questions:**

**Faculty Survey Questions:**

1. In the last 4 weeks, were you the attending on a General Medicine resident service at the University in a one-week or two-week stretches? Note, this does not apply to Newburgh.

- One-week stretch
- Two-week stretch
- Other – please specify

2. Please rate your overall satisfaction with the following aspects of your most recent time on the General Medicine teaching service at the University: (Likert scale of satisfaction - Very Dissatisfied; Somewhat Dissatisfied; Neither Satisfied nor Dissatisfied; Somewhat Satisfied; Very Satisfied)

- Your Clinical skills
- Your interest in teaching
- Your accessibility/availability
- Degree of autonomy provided to resident team
- Degree of supervision provided to resident team
- Patient safety
- Your morale

3. How true do you feel the following statements are about you during the past two weeks?

- Not true at all
- Somewhat true
- Moderately true
- Very true
- Completely true
- I feel happy at work.
- I feel worthwhile at work.
- My work is satisfying to me.
- I feel in control when dealing with difficult problems at work.
- My work is meaningful to me.
- I am contributing professionally (e.g. patient care, teaching, research, and leadership) in the ways I value most.

4. To what degree have you experienced the following?

During the past two weeks I have felt…

- Not at all
- Very little
- Moderately
- A lot
- Extremely
- A sense of dread when I think about work I have to do.
- Physically exhausted at work.
- Lacking in enthusiasm at work.
- Emotionally exhausted at work.

During the past two weeks my job has contributed to me feeling…

- Less empathic with my patients.
- Less empathetic with my colleagues.
- Less sensitive to others’ feelings/emotions.
- Less interested in talking to my patients.
- Less connected with my patients.
- Less connected with my colleagues.

5. Regarding time as an attending on a General Medicine resident service at the University, which of the following best reflects your preference?

- Strongly prefer 1 week
- Somewhat prefer 1 week
- No preference
- Somewhat prefer 2 weeks
- Strongly prefer 2 weeks

6. Why
7. What is your gender identity?

Man

Woman

Non-binary / non-conforming

Prefer to self identify ______________

Prefer not to answer

8. What is your race/ethnicity?

American Indian or Alaska Native

Asian

Black or African American

Hispanic or Latinx

Native Hawaiian or Other Pacific Islander

White

Prefer to self describe	___________

Prefer to Not Answer

9. What division is your clinical appointment in? Select all that apply

- Hospital Medicine – Internal Medicine
- Hospital Medicine – Pediatrics
- Pulmonary
- General Medicine/Primary Care

10. Approximately how much of your FTE is allotted to clinical time in any capacity (e.g. direct patient care and/or rounding). Please round up to the nearest number
11. How many years have you been a faculty? This includes any time prior to coming to Michigan. State a number e.g. 7

**Resident Survey Questions:**

1. In your recent General Medicine rotation at the University, did your faculty supervisor(s) work with you for one-week or two-week stretches? Note, this does not apply to Newburgh.

- One-week stretches
- Two-week stretches
- Other – please specify_____

2. Please rate your overall satisfaction with the following aspects of your most recent time on the General Medicine teaching service at the University: (Likert scale of satisfaction - Very Dissatisfied; Somewhat Dissatisfied; Neither Satisfied nor Dissatisfied; Somewhat Satisfied; Very Satisfied)

- Faculty’s clinical skills
- Faculty’s interest in teaching
- Faculty’s accessibility/availability
- Overall satisfaction with the faculty
- Degree of autonomy
- Degree of supervision
- Patient safety
- Time for learning
- Overall satisfaction with the learning environment
- Faculty’s morale

3. Regarding time you work with an attending/faculty on the General Medicine service at the University, which of the following best reflects your preference?

- Strongly prefer working with faculty for 1 week
- Somewhat prefer working with faculty for 1 week
- No preference
- Somewhat prefer working with faculty for 2 weeks
- Strongly prefer working with faculty for 2 weeks

4. Why?
5. What year are you?

PGY1

PGY2

PGY3

PGY4

6. What is your training program?

Internal Medicine - Categorical

Internal Medicine and Pediatrics

Neurology Prelim

Ophthalmology Prelim

Anesthesia

7. What is your gender identity?

Man

Woman

Non-binary / non-conforming

Prefer to self identify ______________

Prefer not to answer

8. What is your race/ethnicity?

American Indian or Alaska Native

Asian

Black or African American

Hispanic or Latinx

Native Hawaiian or Other Pacific Islander

White

Other	___________

Prefer to Not Answer
