# Supplementary material for: Association of 1‐ versus 2‐week faculty rotations for resident teaching services on patient outcomes, learning environment and burnout
Source: J Hosp Med. 2026 Jul 3;21(11):1263–71. doi: 10.1002/jhm.70389 (PMC13728539; doi:10.1002/jhm.70389)
Supplement: Supplementary file 2 [file JHM-21-1263-s002.docx]

**Supplemental Tables – Professional Fulfillment Index (PFI) Results from faculty**

**Supplemental Table 1: Summary Table of PFI results and Percent of Faculty Meeting Cut-points**

| **Outcome** | **Overall**  N = 47 | **One-week group**  N = 30 | **Two-week group**  N = 17 | **p-value** |
| --- | --- | --- | --- | --- |
| Average Professional Fulfillment Score | 3.04 (0.88) | 3.04 (0.96) | 3.03 (0.76) | 0.52 |
| Number meeting cut point of >3.0 for professional fulfillment | 23 (48.9%) | 16 (53.3%) | 7 (41.2%) | 0.55 |
| Average Burnout Score | 0.81 (0.70) | 0.80 (0.68) | 0.82 (0.76) | 0.82 |
| Number meeting cut point of >1.33 for burnout | 10 (21.3%) | 6 (20.0%) | 4 (23.5%) | >0.99 |

**Supplemental Table 2. Faculty professional fulfillment results based on faculty duration**

| **Outcome** | **Overall**  N = 47 | **One-week group**  N = 30 | **Two-week group**  N = 17 | **p-value***^1^* |
| --- | --- | --- | --- | --- |
| I feel happy at work, Mean (SD) | 2.98 (0.97) | 3.03 (1.00) | 2.88 (0.93) | 0.44 |
| I feel worthwhile at work, Mean (SD) | 3.09 (0.97) | 3.13 (1.04) | 3.00 (0.87) | 0.42 |
| My work is satisfying to me, Mean (SD) | 3.13 (0.97) | 3.03 (1.10) | 3.29 (0.69) | 0.63 |
| I feel in control when dealing with difficult problems at work, Mean (SD) | 2.68 (0.96) | 2.70 (1.02) | 2.65 (0.86) | 0.43 |
| My work is meaningful to me, Mean (SD) | 3.26 (0.97) | 3.30 (1.02) | 3.18 (0.88) | 0.47 |
| I am contributing professionally (e.g. patient care, teaching, research, and leadership) in the ways I value most, Mean (SD) | 3.11 (0.96) | 3.07 (1.05) | 3.18 (0.81) | 0.94 |
| Average of the above: | 3.04 (0.20) | 3.04 (0.20) | 3.03 (0.24) | 0.95 |
| *^1^* Wilcoxon rank sum test; Fisher’s exact test | | | | |

**Supplemental Table 3. Faculty work exhaustion and interpersonal disengagement results based on faculty duration**

| **Outcome** | **Overall**  N = 47 | **One-week group**  N = 30 | **Two-week group**  N = 17 | **p-value***^1^* |
| --- | --- | --- | --- | --- |
| (**Work Exhaustion**) During the past two weeks I have felt… | |  |  |  |
| A sense of dread when I think about work I have to do, Mean (SD) | 0.83 (0.79) | 0.77 (0.68) | 0.94 (0.97) | 0.68 |
| Physically exhausted at work, Mean (SD) | 1.06 (0.96) | 1.13 (0.97) | 0.94 (0.97) | 0.52 |
| Lacking in enthusiasm at work, Mean (SD) | 0.94 (0.82) | 0.90 (0.84) | 1.00 (0.79) | 0.56 |
| Emotionally exhausted at work, Mean (SD) | 0.87 (0.77) | 0.87 (0.78) | 0.88 (0.78) | 0.90 |
| Average for **work exhaustion**: | 0.93 (0.10) | 0.92 (0.16) | 0.94 (0.05) | 0.92 |
| (**Interpersonal Disengagement**) During the past two weeks my job has contributed to me feeling… | |  |  |  |
| Less empathic with my patients, Mean (SD) | 0.70 (0.81) | 0.73 (0.83) | 0.65 (0.79) | 0.75 |
| Less empathetic with my colleagues, Mean (SD) | 0.68 (0.73) | 0.67 (0.71) | 0.71 (0.77) | 0.91 |
| Less sensitive to others’ feelings/emotions, Mean (SD) | 0.70 (0.75) | 0.67 (0.76) | 0.76 (0.75) | 0.64 |
| Less interested in talking to my patients, Mean (SD) | 0.77 (0.89) | 0.77 (0.97) | 0.76 (0.75) | 0.72 |
| Less connected with my patients, Mean (SD) | 0.79 (0.93) | 0.83 (1.02) | 0.71 (0.77) | 0.90 |
| Less connected with my colleagues, Mean (SD) | 0.74 (0.77) | 0.67 (0.76) | 0.88 (0.78) | 0.31 |
| Average for **Interpersonal Disengagement**: | 0.73 (0.04) | 0.73 (0.07) | 0.75 (0.08) | 0.92 |
| Averages for all 10 of the above: | 0.81 (0.12) | 0.80 (0.14) | 0.82 (0.12) | 0.92 |
| *^1^* Wilcoxon rank sum test; Fisher’s exact test | | | | |
