# Supplementary material for: Association of 1‐ versus 2‐week faculty rotations for resident teaching services on patient outcomes, learning environment and burnout
Source: J Hosp Med. 2026 Jul 3;21(11):1263–71. doi: 10.1002/jhm.70389 (PMC13728539; doi:10.1002/jhm.70389)
Supplement: Supplementary file 3 [file JHM-21-1263-s003.docx]

**Supplemental Appendix - 2**

**Faculty and Resident Demographics By Duration of Faculty Rotation**

| **Faculty Demographics** |  |  |  |  |
| --- | --- | --- | --- | --- |
| **Characteristic** | **Overall**  N = 47 | **One-week group**  N = 30 | **Two-week group** N = 17 |  |
| Gender, N (%) |  |  |  |  |
| Female | 14 (29.8%) | 10 (33.3%) | 4 (23.5%) |  |
| Male | 32 (68.1%) | 20 (66.7%) | 12 (70.6%) |  |
| Prefer not to say | 1 (2.1%) | 0 (0.0%) | 1 (5.9%) |  |
| Division(s), N (%) |  |  |  |  |
| General Medicine/Primary Care | 5 (10.6%) | 0 (0.0%) | 5 (29.4%) |  |
| Hospital Medicine - Internal Medicine | 38 (80.9%) | 26 (86.7%) | 12 (70.6%) |  |
| Hospital Medicine - Internal Medicine & Pediatrics | 4 (8.5%) | 4 (13.3%) | 0 (0.0%) |  |
| Clinical Appointment, Mean % full-time effort (SD) | 51.0 (34.6) | 57.9 (36.3) | 39.1 (29.1) |  |
| Non-clinical time, Mean % full-time effort (SD) | 24.6 (27.7) | 15.2 (19.5) | 41.2 (32.3) |  |
| Years have been a faculty, Mean (SD) | 12.8 (10.1) | 8.1 (7.7) | 20.7 (8.9) |  |
| **Resident Demographics** |  |  |  | |
| **Characteristic** | **Overall**  N = 44 | **One-week group**  N = 15 | **Two-week group**  N = 20 | **Mixed-week group**  N = 9 |
| Years of resident, N (%) |  |  |  |  |
| Intern | 27 (61.4%) | 7 (46.7%) | 14 (70.0%) | 6 (66.7%) |
| Resident (PGY2-4) | 17 (38.6%) | 8 (53.3%) | 6 (30.0%) | 3 (33.3%) |
| Training program, N (%) |  |  |  |  |
| Prelim (Anesthesia, Ophthalmology, Neurology) | 11 (25.0%) | 3 (20.0%) | 6 (30.0%) | 2 (22.2%) |
| Categorical Internal Medicine or Med-Peds | 33 (75.0%) | 12 (80.0%) | 14 (70.0%) | 7 (77.8%) |
| Gender, N (%) |  |  |  |  |
| Female | 20 (46.5%) | 6 (40.0%) | 10 (52.6%) | 4 (44.4%) |
| Male | 23 (53.5%) | 9 (60.0%) | 9 (47.4%) | 5 (55.6%) |
